# New Advances in Cardiovascular Drugs: A Celebration of the 90th Birthday of Professor Akira Endo

**DOI:** 10.3390/biomedicines12122716

**Published:** 2024-11-27

**Authors:** Alfredo Caturano

**Affiliations:** Department of Human Sciences and Promotion of the Quality of Life, San Raffaele Roma Open University, 00166 Rome, Italy; alfredo.caturano@virgilio.it; Tel.: +39-3338616985

## 1. Introduction: Professor Endo’s Journey and Revolutionary Discoveries

In this Special Issue, we celebrate a giant of cardiovascular pharmacology, Professor Akira Endo, on the occasion of his 90th birthday. Professor Endo’s journey to improve cardiovascular research was driven by curiosity and a dedication to innovation. Born on November 14, 1933, in Higashiyuri, Northern Japan, he developed a deep interest in the natural world, particularly fungi, which led him to the field of biochemistry. Inspired by the transformative work of Alexander Fleming, Professor Endo pursued his studies at Tokohu University, graduating from the Faculty of Agriculture in 1957 [1]. After completing his Ph.D., he joined Sankyo Pharmaceuticals in Tokyo, where he initially focused on developing fungal enzymes for juice production. However, a turning point came when he theorized that certain fungi might produce compounds that inhibit the enzyme 3-hydroxy-3-methyl-glutaryl (HMG)-CoA reductase—the rate-limiting enzyme in cholesterol biosynthesis. His hypothesis laid the foundation for the development of statins, a class of drugs that would prove essential in lowering cholesterol and reducing cardiovascular risk [1]. In 1971, Professor Endo embarked on this ambitious exploration, sifting through nearly 6000 fungal compounds. His perseverance paid off with the discovery of mevastatin (ML-236B, compactin) in 1973, marking the inception of statin therapy. However, at that time, the significance of this discovery was not immediately apparent. Only in the ensuing years did researchers, including the Nobel laureates Michael Brown and Joseph Goldstein, begin to unravel the role of low-density lipoprotein (LDL) in atherosclerosis, providing context for the groundbreaking nature of Professor Endo’s work [1]. Despite various challenges and skepticism from some sectors, statins gained traction, culminating in the development and eventual approval of lovastatin in the United States in 1987. Since then, statins have become a cornerstone in the prevention of cardiovascular disease (CVD), significantly lowering the risk of myocardial infarction, stroke, and other cardiovascular events.

## 2. The Legacy of Statins and Their Broad Impact

The impact of statins on public health cannot be overstated. Statins have proven invaluable in CVD prevention by lowering cholesterol levels and addressing a primary driver of atherosclerosis. Numerous studies over the last several decades have demonstrated their efficacy in the primary and secondary prevention of cardiovascular events, transforming the treatment landscape for conditions once deemed inexorably progressive [2]. Beyond their cholesterol-lowering effects, statins have shown potential benefits in various other fields, including anti-inflammatory properties, and emerging research has explored possible anticancer benefits [3,4]. This broad therapeutic versatility reflects the profound impact of Professor Endo’s work, which has fundamentally reshaped our understanding and management of cardiovascular risk factors [5,6]. Today, millions of patients benefit from statins and their life-saving properties, and this is directly attributable to Professor Endo’s tenacity and vision. By reducing global cardiovascular mortality and morbidity rates, statins exemplify the ideal of translating scientific discovery into societal benefits, demonstrating the power of curiosity-driven research in tackling some of humanity’s most pressing health issues.

## 3. Expanding Horizons in Cardiovascular Risk Management: From Singular Targets to Multifactorial Strategies

Professor Endo’s pioneering work on statins marked a turning point in cardiovascular medicine, demonstrating the power of targeted pharmacological interventions in lowering cholesterol and, ultimately, reducing the risk of cardiovascular events. However, as the understanding of CVD advanced, so did the recognition that its underlying drivers extend beyond cholesterol alone. Today, cardiovascular risk is understood as a complex, multifactorial process influenced by metabolic, genetic, and environmental factors, as well as lifestyle and social determinants [7]. This broader view has led to a paradigm shift in risk management, where single-target therapies like statins are now combined with additional treatments and approaches that address the diverse contributors to CVD [5,6]. Building on the foundation established by statins, the introduction of novel therapies such as PCSK9 inhibitors, SGLT2 inhibitors, and GLP-1 receptor agonists has further transformed the landscape of cardiovascular prevention. Each of these agents targets distinct mechanisms, from improving glucose metabolism and reducing body weight to providing direct cardiovascular protection, particularly in high-risk groups like those with diabetes and chronic kidney disease [5,6]. This multimodal approach aligns with the current emphasis on precision medicine, where the goal is to provide tailored interventions based on a patient’s unique risk profile rather than a one-size-fits-all solution [8,9,10].

In addition to pharmacological advancements, the importance of lifestyle modifications and social determinants of health has become increasingly clear. Diet, physical activity, smoking cessation, and weight management remain essential pillars of CVD prevention and are now often prescribed alongside drug therapies [11]. This holistic approach underscores the evolving role of clinicians, who must not only prescribe medications but also guide patients in modifying their behaviors to address these lifestyle factors. Public health strategies have also shifted toward more comprehensive risk-reduction models, incorporating community-based interventions that promote healthier living environments, especially in underserved populations, where CVD risk remains disproportionately high [12].

## 4. Overview of Published Articles

Building on Professor Endo’s legacy, the field of cardiovascular pharmacology has continued to evolve, propelled by discoveries that extend and refine our approach to managing CVD. This Special Issue brings together contributions from leading researchers in this field, each exploring a facet of cardiovascular drug development that holds promise for future clinical practice.

Josipa Radić et al. (contribution 1) investigated the relationship between arterial stiffness, measured by pulse wave velocity (PWV), and various factors in a study in hypertensive patients. They found that older patients with poorly controlled blood pressure exhibited higher PWV, influenced by systolic blood pressure and certain medications. Their research highlights the connections between arterial stiffness, blood pressure control, and body composition.

Iida Tuunanen et al. (contribution 2) explored the association between prediagnostic metformin and statin use and the prognosis of patients with hepatocellular carcinoma (HCC) and type 2 diabetes. They found that metformin use was linked to reduced overall mortality (hazard ratio 0.84) compared to no use. The study concluded that while metformin users showed slightly reduced HCC and other-cause mortality, statin use did not significantly impact mortality outcomes in this population.

Jan Kyselovic et al. (contribution 3) investigated outcomes and predictive factors in autologous bone marrow cell (BMC) therapy in patients with “no-option” critical limb ischemia (CLI). They identified baseline C-reactive protein (CRP) and transcutaneous oxygen pressure (TcPO2) as prognostic factors in treatment response, with atorvastatin use associated with improved TcPO2 and reduced pain post-treatment. The study concluded that the prior use of statin and renin–angiotensin system (RAS)-acting agents positively correlated with treatment outcomes, indicating a potential role for these agents in enhancing the efficacy of BMC therapy.

Peter Stanko et al. (contribution 4) investigated the protective effects of angiotensin receptor-neprilysin inhibitors (ARNIs) in a model of hypertension-induced heart disease using Wistar rats. They found that ARNI treatment, similarly to captopril, reduced systolic blood pressure and alleviated left-ventricular (LV) hypertrophy and fibrosis, preventing systolic and diastolic dysfunction. This study concluded that ARNIs may offer significant protection against structural remodeling and functional impairment in hypertensive heart disease.

Nanami Irie et al. (contribution 5) investigated the efficacy of a statin–dipyridamole combination treatment in metastatic melanoma, focusing on its effects in both human and canine melanoma cell lines. They found that combining atorvastatin with dipyridamole significantly reduced the half-maximal inhibitory concentration (IC50) by 68–92%, with some cell lines showing nearly complete inhibition of proliferation. The study concluded that this combination therapy could enhance melanoma treatment, particularly in patients with the BRAF V600E mutation, and suggested potential new developments in melanoma therapy based on the concordant findings in both canine and human models.

Olena Popazova et al. (contribution 6) assessed the cardioprotective effects of nitric oxide (NO) system modulators, including L-arginine, Thiotriazoline, Angiolin, and Mildronate, in newborn rats following intrauterine hypoxia. This study involved 50 female rats, with offspring divided into groups based on treatment after prenatal hypoxia. The results indicated that Angiolin enhanced eNOS mRNA expression and activity in the myocardium, while L-arginine and Thiotriazoline helped restore normal cardiac nitroxidergic system parameters, and Mildronate increased iNOS mRNA levels while reducing nitrotyrosine levels, highlighting its antioxidative properties.

Ying Jie Chee and Rinkoo Dalan (contribution 7) review the increasing incidence of CVD and kidney disease in T2DM and emphasized that a solely glucose-centric approach is inadequate in mitigating cardiovascular risks. They highlight recent advancements in pharmaceutical options, particularly sodium-glucose cotransporter type 2 inhibitors and glucagon-like peptide receptor agonists, which show strong cardiorenal protective effects. The review also discusses emerging combinatorial therapies to enhance patient adherence and improve glycemic control, as well as exploring mechanisms of cardiorenal protection and summarizing upcoming agents in early-phase trials.

Inês Henriques Vieira et al. (contribution 8) review the relationship between T2DM and stroke risk, noting that while strict glycemic control benefits microvascular complications, its role in reducing macrovascular complications such as stroke is less established. They report that current risk reduction strategies focus on multiple factors, including hypertension and dyslipidemia, and that novel T2DM therapies have been the subject of cardiovascular safety trials since 2008. The review highlights the potential stroke prevention benefits of glucagon-like peptide 1 receptor agonists, while the findings regarding sodium-glucose cotransporter type 2 inhibitors remain controversial and dipeptidyl peptidase 4 inhibitors appear neutral regarding stroke prevention.

Andrea Mormone et al. (contribution 9) explored the significance of hypercholesterolemia in the development of lipid plaques, emphasizing the role of elevated low-density lipoprotein cholesterol (LDL-C) in increasing the risk of cardiovascular, cerebrovascular, and peripheral arterial diseases. They report that the control of LDL-C levels is a critical modifiable risk factor in reducing the morbidity and mortality associated with cardiovascular conditions. The review highlights the historical effectiveness of statins, which inhibit the enzyme HMG-CoA reductase, but also introduces newer non-statin agents that offer a favorable cost–benefit ratio as alternative treatments for hypercholesterolemia.

Janette Alejandra Gamiño-Gutiérrez et al. (contribution 10) discuss the significant impact of cardiovascular diseases on global morbidity and mortality. They focus on the renin–angiotensin system (RAS), noting that the overactivation of the classical RAS contributes to disease progression. In contrast, the counter-regulatory RAS produces peptides like Angiotensin-(1-7), which activate cardioprotective mechanisms through receptors such as AT2R, MasR, and MrgD. These mechanisms include reducing cardiac fibrosis, promoting vasodilation, and lowering blood pressure. This review highlights the therapeutic potential of these counter-regulatory peptides in mitigating cardiovascular disease progression.

## 5. The Future of Cardiovascular Pharmacotherapy

As we look to the future, cardiovascular pharmacology stands on the brink of unprecedented advancements. Emerging research into gene editing and RNA interference offers promising avenues for more precise and effective treatments [13]. Gene editing, utilizing technologies like CRISPR-Cas9, allows for the direct modification of specific genes associated with cardiovascular diseases, potentially correcting genetic defects or altering disease pathways at their source [14]. This could lead to targeted therapies that address the underlying causes of conditions such as familial hypercholesterolemia or other genetic disorders impacting cardiovascular health. RNA interference (RNAi) involves the use of small RNA molecules to silence the expression of specific genes. By targeting and degrading messenger RNA (mRNA) associated with disease pathways, RNAi can effectively reduce the production of harmful proteins implicated in cardiovascular conditions. Both gene editing and RNAi hold the potential to revolutionize treatment approaches, offering tailored interventions that align with individual patient profiles. In addition to exploring new mechanisms of action, researchers are increasingly investigating ways of optimizing the delivery and efficacy of existing drugs [14]. These efforts have the potential to provide more personalized, effective care for patients with cardiovascular disease [15,16]. By combining established drugs like statins with newer therapies, we aim to further improve outcomes for high-risk patients, opening up new avenues for the prevention and management of cardiovascular disease. 

## 6. Tribute to Professor Endo and Closing Reflections

Finally, we would like to pay tribute to Professor Akira Endo, whose life’s work exemplifies the profound impact that one individual can have on global health. Although he passed away in June 2024, Professor Endo’s discovery of statins continues to save countless lives and remains an enduring source of inspiration for generations of researchers dedicated to advancing scientific knowledge. His contributions extend far beyond cardiovascular pharmacology, standing as a powerful testament to the transformative power of curiosity, innovation, and perseverance in the pursuit of scientific progress. For instance, statins have not only significantly reduced rates of myocardial infarction and stroke but have also transformed clinical guidelines and practices worldwide, leading to more proactive approaches to managing cholesterol levels and cardiovascular risk factors. Millions of patients currently benefit from statins, showcasing how a single discovery can ripple through healthcare systems, influencing treatment paradigms and improving patient outcomes.

As we celebrate Professor Endo’s legacy on what would have been his 90th birthday, we recognize his influence on the life-saving therapies now accessible to patients worldwide. Looking ahead, we are inspired to carry forward his work, embracing the same spirit of inquiry that propelled his pioneering research. Through ongoing dedication to research and innovation, we strive to honor Professor Endo’s memory by advancing cardiovascular pharmacotherapy. Like a spark igniting a torch, a true teacher leaves aspects of themselves along every path that they illuminate, so that their legacy shines on long after their journey has ended. Professor Endo was such a light, illuminating a path that has profoundly transformed cardiovascular medicine—a path that will continue to guide us toward a healthier future for generations to come.

## Data Availability

No dataset was generated for the publication of this article.
